# Organoids: An Emerging Tool to Study Aging Signature across Human Tissues. Modeling Aging with Patient-Derived Organoids

**DOI:** 10.3390/ijms221910547

**Published:** 2021-09-29

**Authors:** Margalida Torrens-Mas, Catalina Perelló-Reus, Cayetano Navas-Enamorado, Lesly Ibargüen-González, Andres Sanchez-Polo, Juan Jose Segura-Sampedro, Luis Masmiquel, Carles Barcelo, Marta Gonzalez-Freire

**Affiliations:** 1 Vascular and Metabolic Pathologies Group, Health Research Institute of the Balearic Islands (IdISBa), 07120 Palma de Mallorca, Spain; margalida.torrens@ssib.es (M.T.-M.); caye.navas.enamorado@gmail.com (C.N.-E.); poloasp@gmail.com (A.S.-P.); lmasmiquel@hsll.es (L.M.); 2 Translational Research in Aging and Longevity (TRIAL) Group, Health Research Institute of the Balearic Islands (IdISBa), 07120 Palma de Mallorca, Spain; 3 Translational Pancreatic Cancer Oncogenesis Group, Health Research Institute of the Balearic Islands (IdISBa), 07120 Palma de Mallorca, Spain; catalinamaria.perello@ssib.es (C.P.-R.); leslyyaritza.ibarguen@ssib.es (L.I.-G.); 4 General & Digestive Surgery Department, Hospital Universitario Son Espases, 07120 Palma de Mallorca, Spain; segusamjj@gmail.com; 5 School of Medicine, University of the Balearic Islands, 07120 Palma de Mallorca, Spain

**Keywords:** organoids, aging, precision medicine

## Abstract

The biology of aging is focused on the identification of novel pathways that regulate the underlying processes of aging to develop interventions aimed at delaying the onset and progression of chronic diseases to extend lifespan. However, the research on the aging field has been conducted mainly in animal models, yeast, *Caenorhabditis elegans*, and cell cultures. Thus, it is unclear to what extent this knowledge is transferable to humans since they might not reflect the complexity of aging in people. An organoid culture is an in vitro 3D cell-culture technology that reproduces the physiological and cellular composition of the tissues and/or organs. This technology is being used in the cancer field to predict the response of a patient-derived tumor to a certain drug or treatment serving as patient stratification and drug-guidance approaches. Modeling aging with patient-derived organoids has a tremendous potential as a preclinical model tool to discover new biomarkers of aging, to predict adverse outcomes during aging, and to design personalized approaches for the prevention and treatment of aging-related diseases and geriatric syndromes. This could represent a novel approach to study chronological and/or biological aging, paving the way to personalized interventions targeting the biology of aging.

## 1. Introduction: Organoids, Spheroids, and Matrix-Embedded 3D Cultures

Patient-derived organoids (PDOs) are self-organized 3D tissue cultures that are derived from stem cells. Isolated patients’ stem cells differentiate to form an organ-like tissue comprising multiple cell types. Organoids have self-renewal and self-organization capabilities and retain the characteristics of the physiological structure and function of their source [1,2]. Recent culturing advances aim to create the right environment for the stem cells so they can follow their own genetic instructions to self-organize, forming organoid structures that resemble miniature organs composed of many cell types. This approach provides tractable in vitro models of human physiology and pathology, thereby enabling interventional studies that are difficult or impossible to conduct in human subjects [1,3]. Attempts to model the biology of human organs—including the differentiation of human stem cells in 2D, in either the presence or absence of a 3D matrix; bio-printing of human cells; and the culture of cells in a microfluidic device (“organ-on-a-chip”)—were made prior to the emergence of organoids and have shown some potential for drug screening or human disease research.

During the late twentieth and early twenty-first centuries, the use of classical cell lines and animal model systems in biomedical research has helped to improve our understanding of cellular signaling pathways, to identify potential drug targets and to guide the design of candidate drugs for pathologies including cancer and infectious diseases, among others [3,4]. Recent studies have identified biological processes that are specific to the human body, such as brain development, metabolism, and the test of drug efficacy that cannot be modeled in animal or cell models. Nevertheless, extrapolating results from these model systems to humans has become a major bottleneck in the drug discovery process. Therefore, the emergence of human in vitro 3D cell cultures, such as organoids, spheroids, and matrix-embedded 3D cultures has received widespread attention due to the potential to overcome these limitations [4,5,6]. These 3D structures of cultured cells recapitulate important aspects of in vivo organ development and biological function. Such cultures can be crafted to replicate much of the complexity of an organ or to express selected aspects of its physiology like producing only certain types of cells [1,2,3].

Spheroids form by spontaneous aggregation of cells followed by the binding of cell surface integrins to the extracellular matrix (ECM). After initial cell–cell contact, cells upregulate E-Cadherin, which accumulates on the cell surface and then the spheroid becomes a compact structure through strong intercellular E-cadherin interactions [6]. Different spheroid models have been described based on their cellular sources. Multicellular tumor spheroids (MCTS) are often made from cancer cell lines but rarely from tumor tissues. MCTS show little histological resemblance to the original tumor, but they mimic metabolic and proliferation gradients of the in vivo tumor and model clinically relevant resistance to chemotherapy. The advantages of MCTS are that they are clonal, simple to expand into large cultures, and suitable for high-throughput systems [5,6,7].

In contrast, organoids grow from stem cells, which can divide indefinitely and produce different types of cells as part of their progeny. Organoids allow genetic and pharmacological manipulation in a complex cellular context that reflects human biology and enable investigations of the early stages of organ development and disease onset. They complement (and may eventually replace) animal models in many areas of preclinical drug development. Moreover, they provide patient-specific “avatars” for drug development and precision therapies, including treatments for cancer, rare genetic diseases (such as cystic fibrosis), and complex multifactorial disorders (such as epilepsy). Finally, they promise to contribute to regenerative medicine, with the goal of producing functional biological structures that can be transplanted into patients [3,4,5,6,7,8,9].

## 2. Approaches to Generate a Patient-Derived Human Organoid: Surgical Resections, Liquid Biopsy, and iPSC-Derivation

The first organoids were developed from tissues of animal models. However, some biological aspects are unique to humans, and as a consequence, these models show limitations recapitulating human pathology. In that sense, PDOs emerged as a model to study cancer, infectious diseases, and inheritable genetic disorders [10,11,12,13]. The generation of organoids requires the use of stem cells, which can be either (a) pluripotent stem cells (PSCs)—embryonic stem cells (ESC) and induced pluripotent stem cells (iPSCs)—or (b) adult stem cells (ASCs) [7,8,9,13]. For instance, the source of PSC is restricted to iPSCs that are generated through the reprogramming of somatic cells, avoiding the ethical concerns of the use of ESC. iPSCs have the potential to generate all three germ layers. Differentiation into distinct cell and tissue types can be controlled in vitro by the sequential use of different factors that mimic in vivo organ development [7,11]. In contrast, ASCs can be obtained from tissues with regenerative ability, and they have a limited differentiation potential. In that case, the starting material for the generation of the organoids is normal or malignant human tissue that can be obtained from surgical resection or biopsy [10]. In fact, the generation of organoids from these sources allow the expansion and maintenance of this valuable material. The development of the organoids requires the use of specific growth factors depending on the tissue of origin, and they are mainly restricted to the growth of epithelial cells [14]. For that reason, the ASC-derived organoids are less complex than the iPSCs-derived, which might include mesenchymal and epithelial constituents [12]. On the other hand, tissue-derived organoids may recapitulate the genetic and epigenetic signature of the original organ [14], but iPSCs can lose this kind of information due to the dedifferentiation process required for the establishment of the cell line [15], thus hampering the use of iPSCs for preclinical models. In addition, liquid biopsies contain circulating tumor cells (CTCs), which although scarce in material could be good candidates to generate 3D structures, expanding the approaches that could be used to generate organoids [16]. In any case, the procedures to establish organoids rely on the self-renewal and differentiation of tissue-resident stem cells that expand in culture and self-organize into complex three-dimensional structures. Once established, organoids can be initiated from cryopreserved material, cultured using largely traditional cell culture techniques and equipment, and then expanded and cryopreserved for future use [17].

## 3. Organoids as a Model to Study Aging Signature across Tissues

Aging is the major risk factor for most chronic diseases. As a consequence of an increase in lifespan over the years, the elderly population is growing [18]. Frequently, this extension in longevity is not being accompanied with an increase in health-span [19]. Therefore, studying the underlying mechanisms of aging and developing interventions that target the aging process has become a priority field of research for most of the governments and private research agencies worldwide [19].

Organoids might provide a new valuable tool to model the changes that occur during aging across tissues and to study the development of age-associated diseases. Impaired processes and/or damage at the molecular and cellular levels accumulate as we age, leading to a decrease in the reserve capacity or resilience, ultimately developing the aging phenotypes, which have been divided into four domains: body composition, energetic imbalance between availability and demand, homeostatic dysregulation, and neurodegeneration [18,19,20]. Genomic instability, telomere attrition, epigenetic alterations, loss of proteostasis, deregulated nutrient sensing, mitochondrial dysfunction, cellular senescence, stem cell exhaustion, and altered intercellular communication have been identified as important hallmarks of aging in mammals [20]. Modeling these hallmarks using organoids seems to be possible (Table 1).

For instance, aging is associated with a progressive loss of muscle mass and strength and a decline in neurophysiological functions, due to a gradual loss of motor neurons [47]. Recently, neuromuscular 3D organoids in vitro have been developed successfully to examine the roles of human autoantibodies in the pathogenesis of myasthenia gravis [48]. Therefore, with this model we could test whether changes in the neuromuscular junction precede or follow the decline of muscle mass and strength associated with aging. One of the most common approaches to study aging with organoids is deriving organoids from young and old donors. Reduced organoid formation efficiency has been described for aged mice and humans compared to their younger counterparts, which was associated with stem cell dysfunction and epigenetic changes resulting in a reduction in Wnt signaling [21,22,23,24,25]. Aged organoids also show increased levels of senescence markers such as p21 and p16, as well as decreased DNA methyltransferases [21]. Fasting and calorie restriction have been proposed as an anti-aging strategy and have been validated in organoids. Mihaylova et al. [25] showed that fasting for 24 h increased organoid formation and self-renewal potential of organoids derived from old mice. Similarly, improved organoid formation efficiency was observed in calorie-restricted mice, identifying the mTORC1 signaling in Paneth cells [28] and SIRT1 in intestinal stem cells [29,30] as key pathways to ameliorate stem cell function. On the other hand, long-term inflammation has also been modeled in intestinal organoids, and NF-κB signaling has been proposed as a driver of cellular transformation [31].

Skin equivalents (SE) consisting of a 3D culture of fibroblasts and keratinocytes have been developed to study skin aging, either by inducing senescence in vitro or by isolating cells from aged donors. SE have successfully modeled some features of skin aging such as decreased ECM synthesis, cellular loss, and thinner epidermal layer [35]. SE derived from aged donors show some of these histological features of aging [36,38], and p16 has been identified as a driver of these changes, as demonstrated by the modulation of p16 levels in young- and old-derived SE [36]. Extended cultures of SE also recapitulate normal skin aging, including p16 induction [37]. Moreover, Metral et al. [38] showed that the addition of adipose-derived stem cells delayed the expression of senescence markers in SE. SE have also been used to analyze the role of the microenvironment in age-associated changes in the skin. Collagen fragmentation has been described as an important factor for skin aging. Aged fibroblasts detach from the ECM, resulting in impaired TGF-β signaling and ultimately leading to a decrease in the synthesis of ECM components [39,49], further aggravating skin aging and fragility. Senescent melanocytes contribute to skin aging by inducing telomere damage and decreased proliferation of keratinocytes in a paracrine manner [50], while senescent fibroblasts induce changes in the secretome of SE, increasing IL-6, IL-1α, and granulocyte macrophage colony-stimulating factor levels, which contribute to a decreased epidermal layer [51]. ECM age-associated changes have also been analyzed with tendon-derived organoids. Yan et al. [40] observed decreased organoid formation efficiency in organoids from aged donors, lower cell density, and decreased matrix deposition. These changes were associated with higher levels of the senescent markers p21 and p16 and stem cell dysfunction.

Some reports show that human intestinal organoids preserve their original DNA methylation pattern and their epigenetic age when cultured in vitro [26,52]. Notably, Lewis et al. [26] found that the epigenetic age of colon-derived organoids matched the actual age of the donor, while organoids derived from the small intestine showed a slight decrease in epigenetic age. Moreover, cultured mouse colon organoids seem to recapitulate age-associated epigenetic changes [27], opening the possibility of using organoids as a plausible model to study changes in DNA methylation patterns with aging. A lower organoid formation efficiency was also observed in organoids derived from aged alveolar epithelial type II cells. Furthermore, fibrotic cells and cells with shortened telomeres also decreased the organoid formation rate, which was associated with increased senescence and decreased stem cell function due to the activation of the Wnt signaling pathway [41]. Organoids have also been used to model age-associated diseases. For instance, 3D cultures have been developed to study osteoarthritis [53], macular degeneration [54], uterine leiomyoma [55], or Alzheimer’s disease [56], which will be discussed in the next section. Before organoids can become important aging models, some issues must be considered such as the lower efficiency found when reprogramming cells to form organoids from aged donors compared to young ones or the increase in senescence rate when cultured for long time [57,58,59].

## 4. Application of Organoids in Age-Related Diseases: Cancer, Alzheimer’s, and Parkinson’s Disease

As we have stated along the review, organoids have emerged as an invaluable tool in biomedical research and have been extensively developed in the cancer field [60,61]. Other age-related diseases, such as Alzheimer’s Disease (AD) and Parkinson’s Disease (PD), could benefit from this technology.

### 4.1. Cancer

Cancer is considered an age-related disease because its incidence might be explained by the combination of (a) the accumulation of mutations in tissues throughout life and (b) the alterations of the tissue microenvironment that play a role as a selective pressure over them [62]. Moreover, the technical developments primarily achieved in organoid-based cancer research paves the way to the study of other age-related diseases by a patient-derived organoid approach [63]. In that sense, the expansion of clonal organoids from a single stem cell generates distinct mutational signatures, which allow the study of tumor genomic evolution [61]. Blokzijl and colleagues assessed the mutation accumulation in ASCs of the colon, the small intestine, and the liver, showing similar rates among them (36 mutations per year) but with tissue-specific mutational profiles and different cycling rates [32].

Once the candidate driver mutations are described, organoids have the potential to functionally validate them. Thus, wild-type organoids can be genetically engineered to model cancer initiation and progression recapitulating the oncogenic process. CRISPR/Cas9 technology was first used to introduce mutations in the most frequently mutated genes in colorectal cancer (*APC*, *P53*, *KRAS*, and *SMAD4*) in human small intestinal organoids in order to recapitulate the development of colorectal cancer [33]. Similarly, the knockout of tumor suppressor genes can recapitulate tumorigenesis in liver and breast human-derived organoids [34,42]. Interestingly, organoids can also, in part, reproduce the growth factor dependency of tumor cells within their microenvironment. With this approach, the genotype–phenotype correlation of the growth factor dependency was described in gastric cancer and pancreatic tumors allowing the description of tumor subtypes [43,44]. Finally, it is known that a link exists between cancer and infectious agents to which we can be exposed during our lifespan. Thus, the co-culture of organoids with pathogens renders the opportunity to mimic the host–pathogen interactions and pathogenic-induced oncogenesis. In this regard, human gastric organoids were microinjected with *Helicobacter pylori* to model the role of the microorganism in epithelial signaling and proliferation [64]. Recently, the role of human papillomavirus (HPV) in carcinogenesis was studied in ecto- and endocervical-derived organoids [65]. Besides, organoids can be co-cultured with microbiota [66], which is relevant due to its role in cancer and other aging-related processes [67].

### 4.2. Alzheimer’s Disease and Parkinson’s Disease

Even though Alzheimer’s Disease (AD) is one of the leading causes of death worldwide, especially in late life, to date there are currently no available drug treatments to cure the disease. Similarly, Parkinson’s Disease (PD) is the second most frequent neurodegenerative disorder after AD. Organoid systems based on human pluripotent stem cells (hPSCs) and neural stem/precursor cells (NSCs) have shown a promising potential to model neurodegenerative diseases, including AD and PD [45,68,69,70]. 3D brain organoid systems generated from hPSCs can recapitulate important features of AD pathophysiology, such as amyloid plaques and neurofibrillary tangle-like structures [46,68]. Similarly, NSCs utilized to derive human midbrain-specific organoids (hMO) show abundant neurons with dopaminergic identity, thus electrophysiologically functional neurons, as well as astroglia and oligodendrocyte differentiation [70]. Most of the reports using brain organoids in PD or AD have been focused on genetic risk factors, relying on CRISPR/Cas9 gene editing. In both cases, these organoids models have been used to test the efficacy of pharmacological agents in disease progression [45,69].

## 5. Precision Medicine: Using Organoids Systems as a Tool to Screen Anti-Aging Drugs/ Patient-Specific Drug Testing

Precision medicine, or “personalized medicine,” has emerged as an approach for disease treatment and prevention adapted to individual variability and personal characteristics allowing the ability to predict or find the best treatments for a particular condition or disease [71]. This approach requires the integration of all the clinical data, including all the “omics” and molecular information as well as the environment and lifestyle for each individual. Although this will be the future, the use of precision medicine on a daily basis in healthcare is almost restricted to some clinical fields, specifically cancer research, which uses this approach for patient-specific tumor drug testing. Creating comprehensive collections of organoid biobanks might be useful in the near future as a tool to validate candidate drug efficacy and safety as well as to support pre-clinical studies aimed at personalized medicine [72].

Despite advances in the treatment of age-related diseases, the burden of deaths remains high. A key limitation in age-related diseases treatment is the lack of valid predictive biomarkers, which reduces the efficacy of treatments. As in other diseases like cancer, gerontologists are largely unable to predict treatment response for individual patients, resulting in patients receiving ineffective treatment with unnecessary exposure to toxic side effects and high treatment costs. Effective predictive biomarkers are needed to enable personalized medicine and increase survival for patients [73]. Personalized medicine strategies include protein-based, RNA-based, and genome-based stratification, though in oncology, precision medicine has been largely based on genomic biomarkers [74]. However, less than half of patients are eligible for genetically matched treatment, and, for the majority of anticancer agents, no genetic markers are available. A promising predictive biomarker is individualized tumor response testing using PDOs, in which anticancer agents are screened ex vivo on PDOs to predict the clinical response. PDOs represent a superior preclinical model system compared to previous models through their inherent heterogeneity, long-term stability, applicability for high throughput screens, and enhanced capacity to capture tumor characteristics [73,74].

**Figure 1:**
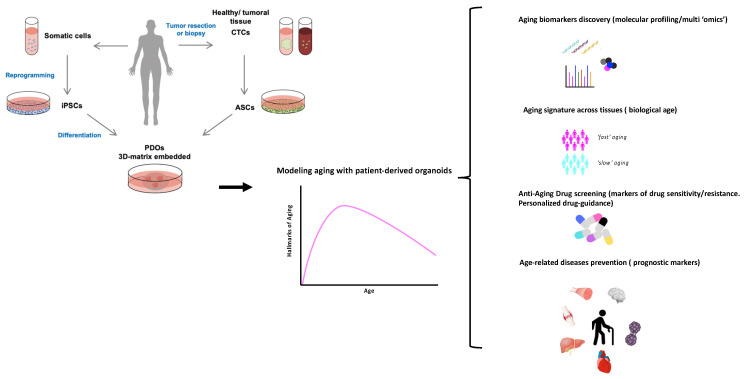
Patient-derived organoids (PDOs) as a personalized aging tool. PDOs can be obtained from reprogramming adult stem cells into uncommitted induced pluripotent stem cells (iPSCs), which through a series of differentiation steps result in the generation of the desired tissue type organoid. Alternatively, tumor resection or liquid biopsy/circulating-tumor cells (CTC) can be the source of the adult stem cell (ASCs) required to establish the organoid. Modeling aging with PDOs have a tremendous potential as a preclinical model tool to discover new biomarkers of aging, to predict adverse outcomes during aging, and to design personalized approaches for prevention and treatment of aging-related diseases and geriatric syndromes. This could represent a novel approach to study chronological and/or biological aging, paving the way to personalized interventions targeting the biology of aging.

In this regard, the description of potential aging-related biomarkers such as telomere length, DNA damage, mitochondrial dysfunction, reactive oxygen species, autophagy, and epigenetic marks [20] opens the possibility to use PDOs as a predictive tool of disease outcome. In addition, the discovery and validation of new aging-related biomarkers using PDOs is a promising field. PDOs could be exposed to panels of clinically relevant anti-aging drugs in order to identify better treatment schemes in bench-to-bedside approaches (Figure 1).

For the past decades several compounds have been found to delay the onset of age-related diseases and increase health-span and lifespan [18,19,75] These compounds target several pathways such as growth factor and insulin signaling pathways (e.g., mTOR inhibitors), carbohydrate and fat metabolism (e.g., metformin), NAD+-dependent sirtuins and NAD pathways (e.g., resveratrol, sirtuins, and NAD2), autophagy (e.g., spermidine), and senescence (e.g., senolytics and senomorphics). Some of these drugs are currently used to treat other age-related diseases such as cancer (senolytics and mTOR inhibitors) or type 2 diabetes (metformin), but they have recently been proposed also as “anti-aging” drugs [75]. Since organoids recapitulate the histological architecture of human tissues in vivo, they can also be used to test potential anti-aging drugs that target one or more of the domains and hallmarks of aging. This could also help to identify the molecular signatures related to treatment efficacy and toxicity. To date and to our knowledge, the specific use of these drugs in organoids with the main goal to directly address the aging mechanism in the absence of disease has not yet been sufficiently studied.

Aging has been associated with a decrease in NAD+ levels [76,77]. NAD+ precursors such as nicotinamide mononucleotide (NMN) and nicotinamide riboside (NR) have been proposed as potential anti-aging drugs [78]. Aged intestinal organoids treated with NMN showed improvement in cell proliferation and a decrease in senescence markers [21]. Similarly, NR treatment improved organoid formation from crypts isolated from old mice through activation of SIRT1 [30]. NMN has also been tested in cerebral organoids derived from AD patients with different genetic backgrounds. Some organoids showed an increase in mitophagy, while others showed mitochondrial biogenesis induction. In this study, an inhibitor of the unfolded protein response, ISRIB, also showed potential as a drug to protect against proteotoxic stress [79]. Some reports also tested different well-known drugs in skeletal muscle organoids and validated this model to screen potential drugs to treat age-associated sarcopenia [80,81]. Senolytic drugs, drugs that eliminate senescent cells, have also been evaluated with organoids. Navitoclax, also known as ABT-263, is an anti-cancer drug that has senolytic activity. It successfully removed senescent cells in a model of uterine leiomyoma in a time-dependent manner [55]. Fisetin, another senolytic compound, has been found to decrease the levels of IL-6 and TNF-α, which have been involved in inflammaging (age-related inflammation) [82], in skin models [83]. Lammermann et al. [84] tested a plant extract with senolytic properties in SE and prevented the effects of the senescence-associated secretory phenotype (SASP). Similarly, the senolytic compound ABT-737 also blocked the effects of the SASP, while mitoQ prevented telomere damage and senescence induction in SE [50]. Another tested anti-aging compound is the synthetic jasmonic acid derivative LR2412, which increased ECM proteins deposition in SE, improving some of the aging effects on the skin [85].

## 6. Limitations, Challenges, and Future Prospects

Even though organoids could potentially become relevant aging models, there are some issues that need to be addressed. Some reports show a lower efficiency of reprogramming cells to form organoids from aged donors compared to young ones, and reprogramming cells from an aged donor into iPSCs may erase some aging epigenetic marks [57,58]. This would introduce unacceptable bias when performing side-by-side experiments. Another important limitation is the lack of a standardized protocol to establish organoids for many organs, which inevitably leads to high variability of this system from one lab to another. Moreover, it is important to obtain stable organoids resembling the adult, mature tissue to study aging. However, a protocol for long-term maintenance of organoids, especially for those derived from non-epithelial sources, is yet to be developed and accepted. Notably, it has been described that mesenchymal stem cells show accelerated senescence when cultured for an extended time [59].

Other limiting factors are the absence of a physiological niche, namely, innervation and vascularization of organoids. Some important physiological functions are under the control of the nervous system. Thus, for some applications, such as modeling normal function and disease, organoids need to include innervation. Some authors have successfully generated innervated organoids [86,87], and several strategies are being studied [88]. On the other hand, the intricate architecture of veins and arteries is difficult to include in the organoid culture; although some studies are currently underway [89]. Overcoming this issue would allow the co-culture of several types of organoids in order to mimic a complex tissue organization and even multi-organ systems for the study of inter-organ communication, including the role of hormones and cytokines that are known to be involved in the aging process.

Furthermore, some culture conditions may favor the selection of certain cell types, and in the case of tumor organoids, cellular clones that are able to survive in this environment. The effect of unintended clonal selection when establishing the organoid would hamper the reproducibility of the experiments and hinder precision medicine applications. In this regard, culture conditions must be closely monitored and results should be replicated in multiple clones per experiment. On the other hand, the microenvironment is usually absent in organoid culture, and thus, cell–stromal interaction is missing. In this sense, the extracellular matrix composition, specific cell types, and the microbiome, which can be critical for tissue function, are still to be defined to better model the complexity of the studied tissue. Finally, not all tissues have been successfully engineered into organoids, as some cell types are difficult to obtain and culture in vitro, especially those deriving from a mesenchymal origin. Moreover, organoid culture is costly, and generating a comprehensive collection representative of a patient’s complexity could be very time-consuming, which hinders the use of organoids for clinical and translational applications.

Despite all the caveats, we believe that in the forthcoming era of precision medicine, modeling aging with patient-derived organoids will help to find biological biomarkers that could capture the inter-individual variability of biological processes of aging before it becomes clinically detectable.

## Figures and Tables

**Table 1:** Summary of studies using organoids to model aging.

Type of Organoid	Addressed Hallmark of Aging	Main Findings	Reference
Gut	Stem cell exhaustion; deregulated nutrient sensing	Lower O.F.E.; altered crypt formation	[21,22,23,24,25]
	Epigenetic changes; cellular senescence	Increased senescence markers; altered DNA methylation	[21,26,27]
	Stem cell exhaustion; deregulated nutrient sensing	CR increased O.F.E.; reduced mTOR signaling	[28,29]
	Stem cell exhaustion; deregulated nutrient sensing	NR supplementation increased O.F.E.	[30]
	Altered intercellular communication	Chronic inflammation led to NF-κB activation and cellular transformation	[31]
	Genomic instability	Tissue-specific mutational profile; tumor development	[32,33]
Liver	Genomic instability	Tissue-specific mutational profile; tumor development	[32,34]
Skin	Cellular senescence; altered intercellular communication	Increased senescence markers; decreased ECM synthesis	[35,36,37]
	Cellular senescence; altered intercellular communication	Adipose stem cells prevent skin senescence	[38]
	Altered intercellular communication	Altered TGF-β/Smad signaling	[39]
Tendon	Stem cell exhaustion; cellular senescence; altered intercellular communication	Lower O.F.E; decreased ECM synthesis; increased senescent markers	[40]
Lung	Stem cell exhaustion; cellular senescence; telomer attrition	Lower O.F.E; shortened telomeres; increased senescent markers	[41]
Breast	Genomic instability	Tumor development	[42]
Gastric	Genomic instability; epigenetic changes; altered intercellular communication	PDO characterization; altered Wnt signaling	[43]
Pancreatic	Genomic instability; altered intercellular communication	PDO characterization; altered Wnt signaling	[44]
Brain	Loss of proteostasis	Amyloid plaques and tau aggregates	[45,46]

O.F.E.: organoid formation efficiency; CR: calorie restriction; NR: nicotinamide riboside; ECM: extracellular matrix; PDO: patient-derived organoids.

## Data Availability

Not applicable.
